# A Double-Layer Patch Antenna for 5–6 GHz Wireless Communication

**DOI:** 10.3390/mi13060929

**Published:** 2022-06-11

**Authors:** Xue-Ping Li, Ming-Rong Ma, Qi-Meng Zhang, Lin Yan, Chang-Qing Wang, Wei Li

**Affiliations:** 1 College of Electronic and Electrical Engineering, Henan Normal University, Xinxiang 453600, China; mrma2020@126.com (M.-R.M.); zqm010628@163.com (Q.-M.Z.); linyan20022022@126.com (L.Y.); 021039@htu.edu.cn (C.-Q.W.); 2 Henan Key Laboratory of Optoelectronic Sensing Integrated Application, Henan Normal University, Xinxiang 453600, China; 3 Henan Engineering Laboratory of Additive Intelligent Manufacturing, Henan Normal University, Xinxiang 453600, China

**Keywords:** microstrip patch antenna, double-layer, wireless local area network, surface current

## Abstract

This paper proposes a compact double-layer microstrip patch antenna with a wide bandwidth of 4.83–6.1 GHz and a gain reaching 4.7 dBi. By folding its mirror image through the electric field symmetry principle of the microstrip antenna, its electrical properties are maintained, and the physical size is halved to the compact size of only 25 × 40 mm^2^. The proposed antenna has the radiation characteristics of a planar inverted-F antenna (PIFA), which can generate the first resonant frequency and realize omnidirectional radiation characteristics. By coupling and feeding the upper patch, the second resonant frequency of the proposed antenna is produced and the directional radiation characteristics of the microstrip patch antenna can be achieved. The consistency of the results between the simulation and test indicates that the proposed antenna design is an ideal potential choice for home wireless local area network (WLAN) communication.

## 1. Introduction

At present, the commercial and industrial demand for wireless local area network (WLAN) communications is increasing. WLAN can provide high-speed connectivity and easy access to networks without wiring. However, the frequency bands of WLAN used in different countries and regions have certain distinctions. In order to connect to local WLAN networks in different regions of the world, it is necessary to design 5 to 6 GHz working frequency antennas [1]. Monopole antennas and related arrays have been used for the application of WLAN frequency bands, but their large size restricts rapid development [2,3]. Microstrip antennas have attracted more attention because of their light weight, small size, and easy manufacturing [4,5]. However, the bandwidth of the microstrip antenna is relatively narrow. There are many methods to increase the bandwidth. An attempt was made to increase the bandwidth by using slot antennas. The shapes of these slots are different, which can produce distinct performances [6]. Radiation patches are E-shaped, H-shaped, E-H-shaped, W-shaped, U-shaped, etc., all of which show good performance when widening the bandwidth [7,8,9,10]. Different feeding methods can be used to increase the bandwidth of the microstrip antenna, such as microstrip line feeding, adjacent coupling feeding, and coaxial probe direct feeding [11,12]. Multi-layer antenna stacking is also very helpful in increasing the bandwidth and achieving omnidirectional characteristics [13,14]. Placing a low dielectric constant at the maximum electric field can also effectively increase the bandwidth of the microstrip antenna [15,16]. Most microstrip antennas work at half the wavelength, and the radiation patch of the microstrip antenna is short-circuited with the ground layer, so that the antenna can resonate at a quarter wavelength, which can significantly reduce the size and increase the bandwidth [17,18,19,20].

This paper proposes a microstrip patch antenna applied in the frequency band of WLAN communication. The working band covers the frequency range from 4.83 to 6.1 GHz. The antenna consists of an upper E-shaped radiating patch and a lower slotted radiating patch with two substrates sandwiched between them. The overall structure of the antenna has the radiation characteristics of a planar inverted-F antenna (PIFA), which can generate the first resonant frequency and realize omnidirectional radiation characteristics. Using the coupling and feeding of the two radiating patches, the second resonant frequency can be produced, and the directional radiation characteristic of the microstrip patch antenna is achieved. Then, the full-space radiation characteristics can be obtained in the entire working frequency band. The parameters of the proposed antenna are optimized with HFSS. In addition, the proposed antenna is made and measured, and the measured results are in good agreement with the simulated results.

## 2. Design and Analysis

### 2.1. Antenna Design

**Figure 1:**
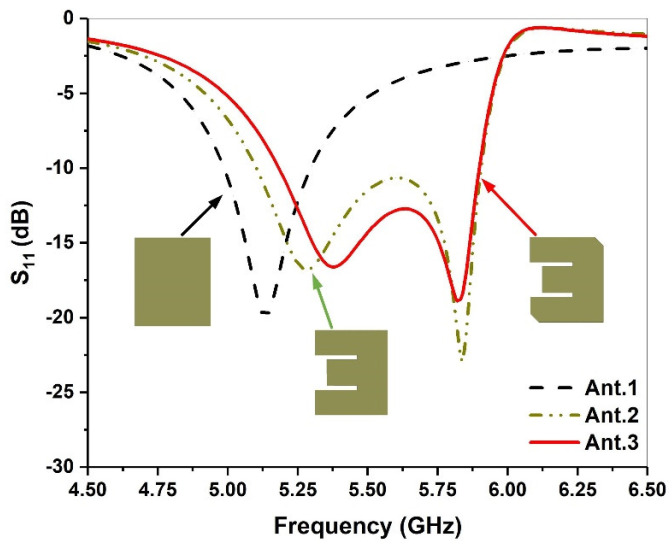
Simulated return loss with different antenna shapes.

**Figure 2:**
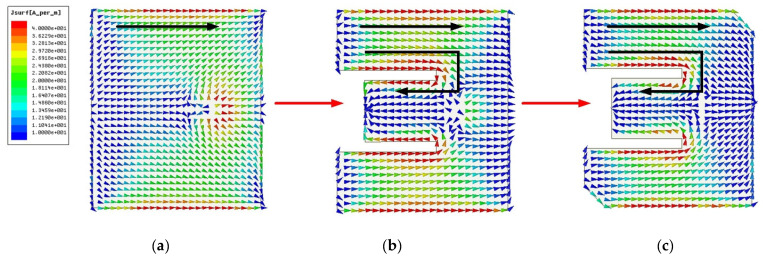
Antenna evolution process: (**a**) Ant.1; (**b**) Ant.2; (**c**) Ant.3.

Figure 1 shows the S-parameters of different radiation patches during the evolution of the antenna. The geometry of the traditional microstrip antenna is a simple rectangular patch. It is directly fed by a coaxial probe, and the surface current is shown in Figure 2a. The resonant frequency is controlled by the length and width, and impedance matching can be achieved by varying the feed location of the coaxial probe. The traditional microstrip antenna has only one resonant frequency, and its bandwidth is narrow. To increase the bandwidth of the microstrip antenna and further realize its miniaturization, a parallel slot is etched in the traditional rectangular patch antenna to change the current path. Then, a second resonant frequency is generated, as shown in Figure 1 and Figure 2b.

From Figure 2b, we observe that the current distribution at the edge of the E-shaped patch is the same as that of the traditional rectangular patch (see Figure 2a), and the first resonant frequency is generated. With the current flowing around the parallel grooves of the E-shaped patch, a new current path has been added. Thus, the second resonant frequency is produced and the bandwidth is expanded, as shown in Figure 1. To further increase the current path length and achieve impedance matching, an E-shaped slot with a truncation angle is introduced, as shown in Figure 2c.

### 2.2. Theoretical Analysis

**Figure 3:**
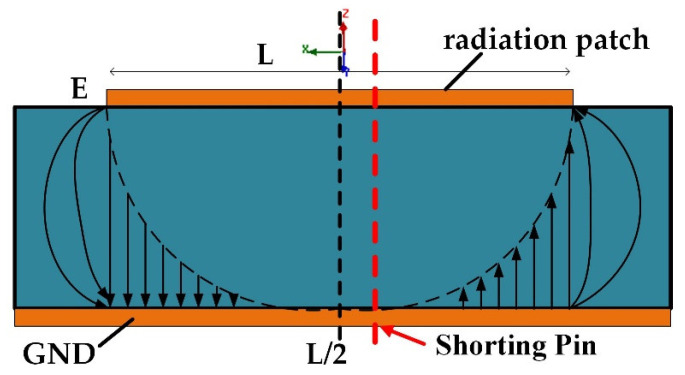
Schematic diagram of microstrip patch antenna radiation.

To miniaturize the microstrip patch antenna, it has been folded. According to the radiation principle diagram of the microstrip patch antenna, as shown in Figure 3, the rectangular patch antenna has zero voltage and electric field at *L*/2, which is equivalent to a short circuit. The short-circuit surface is replaced by a metal column, so that the original resonant frequency remains unchanged. The length of the antenna is reduced from *λ*_0_/2 to *λ*_0_/4 (*λ*_0_ is the wavelength of free space), the electrical size of the antenna remains unchanged and the physical size is halved. Meanwhile, the dual-resonance, wide-bandwidth characteristics are obtained. For the E-shaped patch antenna, the surface current changes, and the electric field zero point is offset. It is noted that the proposed antenna has added a short-circuit column to the red line on the right of *L*/2.

**Figure 4:**
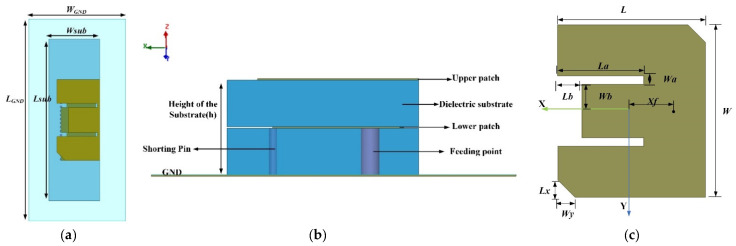
Geometry of the antenna: (**a**) top view; (**b**) side view; (**c**) top view before folding.

The structure of the proposed antenna is demonstrated in Figure 4a,b. To ensure the integrity of the current path in the upper radiating element, the upper layer patch is slightly larger than *L*/2 when folded. The lower patch is excited with a coaxial probe and the upper radiating patch is fed through coupling. By varying the feed location and the distance between the double-layer radiating elements, the impedance matching is realized and the coupling of the two resonant frequencies is completed. Meanwhile, the impedance bandwidth can be expanded. Figure 4c represents the top view of the proposed antenna before folding, and the positions of the parallel slots and truncated corners can be seen. The values of all parameters are shown in Table 1. *Wa* and *La* are the width and length of the parallel grooves, respectively. *Wb* and *Lb* represent the size of the central cut. The thickness of the proposed antenna is *h* and the position of the feeding point is *Xf.*

## 3. Parametric Study

**Figure 5:**
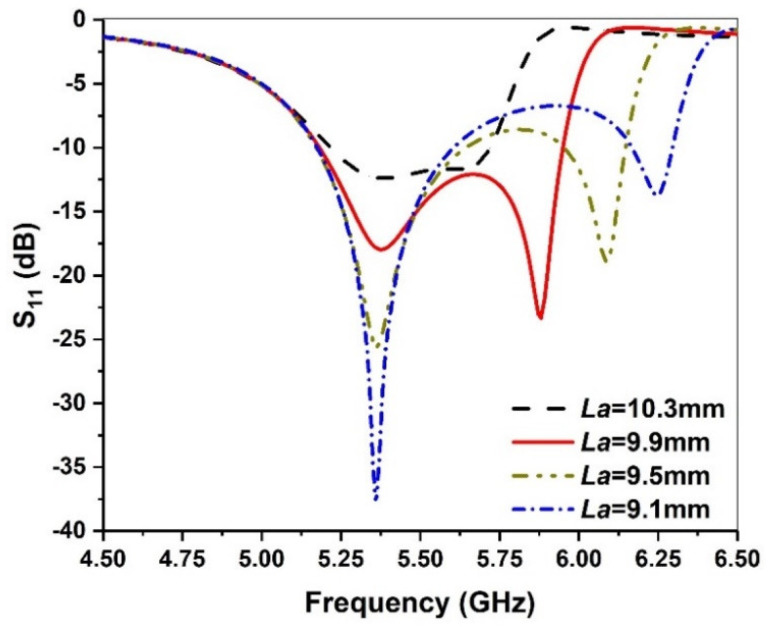
Simulated return loss against frequency for various *La* values.

Change in the length of the parallel slot *La* can change the current around the slot, which affects the second resonant frequency. Figure 5 shows the effect of *La* on return loss. It can be seen that as the length of *La* increases, the higher resonant frequency moves closer to the lower frequency. In addition, if the two resonant frequencies are too far away, the impedance matching of the circuit cannot be good and the reflection coefficient is bad. However, if the two resonant frequencies are too close, the reflection coefficient exhibits a decreased trend, resulting in a reduced overall frequency bandwidth. Therefore, 9.9 mm is selected as the optimal value for the length of the parallel grooves.

**Figure 6:**
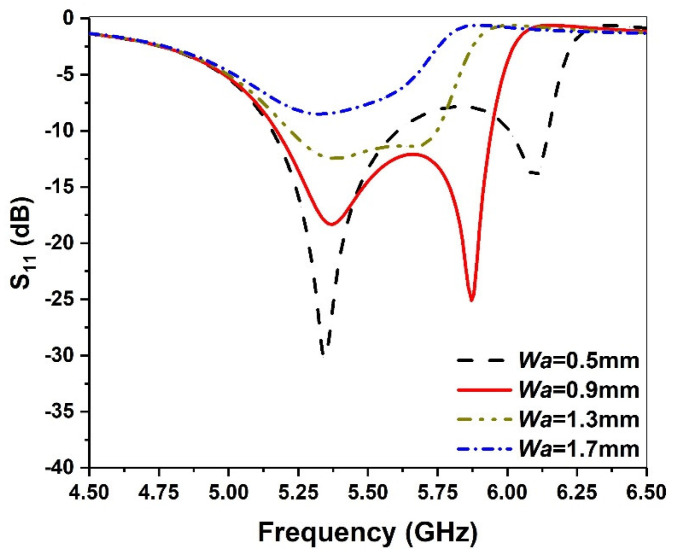
Simulated return loss against frequency for various *Wa* values.

To evaluate the effect of slot width *Wa*, the size is varied from 0.5 mm to 1.7 mm with an interval of 0.4 mm. The corresponding result is depicted in Figure 6. We can see that when *Wa* increases, the second resonant frequency decreases, while the first resonant frequency remains unchanged. If *Wa* is too large, the two resonant frequencies are too close, and the bandwidth demonstrates a reduced trend. Moreover, if *Wa* is too small, the two resonant frequencies are too far away, and |S_11_| of the lower frequency is deteriorated. Therefore, 0.9 mm is chosen as the optimal value of *Wa*.

**Figure 7:**
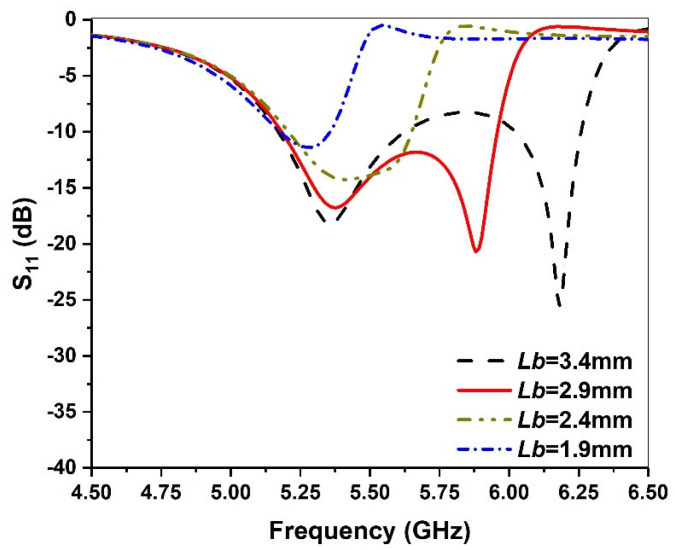
Simulated return loss against frequency for various *Lb* values.

The intercepted part in the middle affects the local current path, thereby introducing a local capacitance effect. In Figure 7, we find that as *Lb* increases, the second resonant frequency moves to a high frequency, while the first resonant frequency remains almost unchanged. Thus, 2.9 mm is chosen as the final value of *Lb*. It is notable that a change in *Wb* has similar behavior to *Lb* and, therefore, is not discussed in this paper for the sake of brevity.

**Figure 8:**
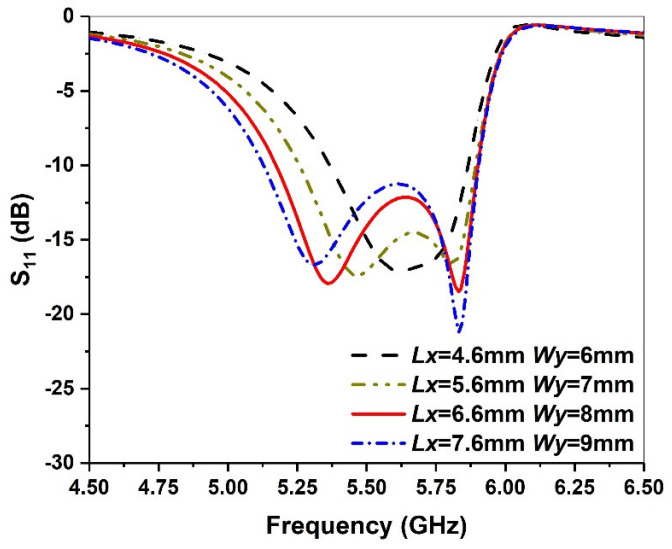
Simulated return loss against frequency for various *Lx* and *Wy* values.

The size of the chamfer can further increase the current path and affect its first resonant frequency, while the second resonant frequency remains unchanged, as shown in Figure 8. To increase the bandwidth, the value of the return loss S_11_ between the two resonant frequency points should be less than −10 dB. Thus, we choose 6.6 mm and 8 mm as the optimized values of *Lx* and *Wy*, respectively.

**Figure 9:**
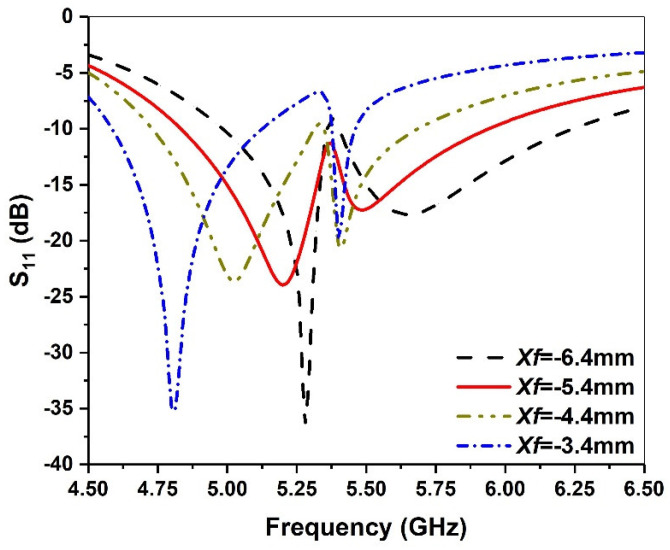
Simulated return loss against frequency for various *Xf* values.

When the antenna is folded, the antenna’s inductive reactance increases, which can induce a bulge in the middle of the two resonant frequencies. By choosing a suitable feed point position, it will be easy to regain the impedance matching between the antenna and the feeder. As shown in Figure 9, when the position of the feeding point is varied from −3.4 mm to −6.4 mm, the first resonant frequency significantly moves to a high frequency, while the second resonant frequency changes very little. When the feed point *Xf* = −5.4 mm is selected, good impedance matching can be achieved.

## 4. Results and Discussion

### 4.1. Impedance Bandwidth

**Figure 10:**
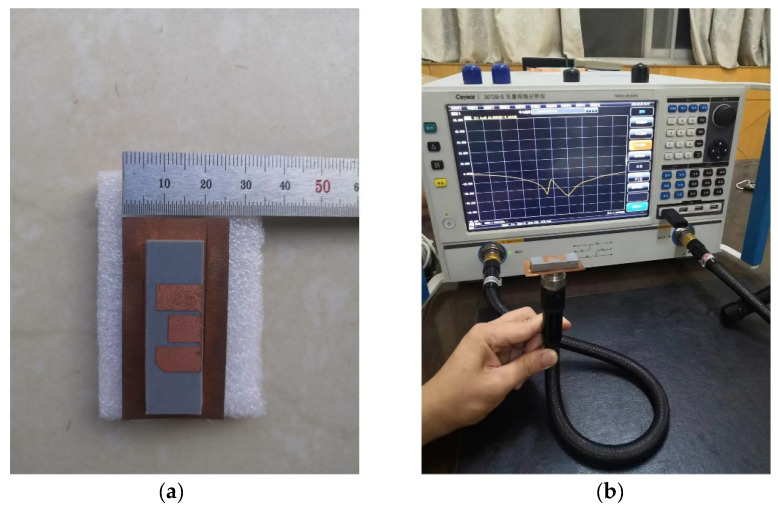
Constructed antenna model: (**a**) actual measurement; (**b**) measured photograph.

**Figure 11:**
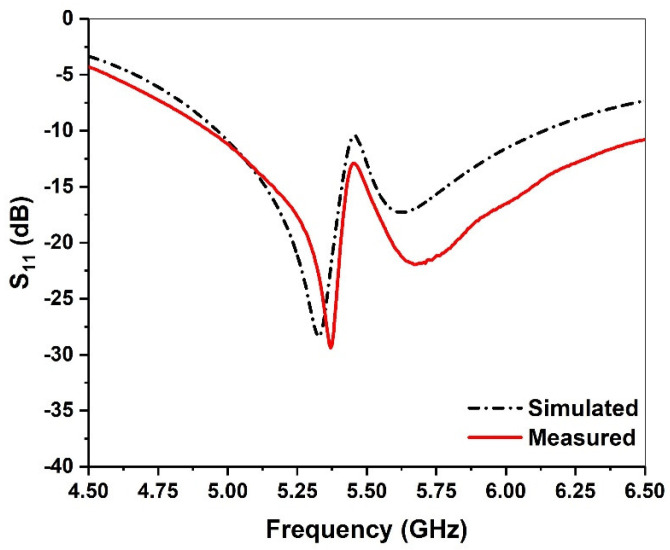
Simulated and measured return loss of the antenna.

To verify the performance of the designed antenna, an actual antenna was fabricated as shown in Figure 10a. The single-layer microstrip patch is short-circuited with a metal pillar at the place where the electric field is zero, and the right half is reserved as the lower patch. In order to ensure the continuous current path of the upper patch, which is slightly larger than the left half of the original microstrip patch antenna, the lower patch is excited with a coaxial probe and the upper radiating patch is fed through coupling, which can realize wide a bandwidth and reduce the surface area. A vector network analyzer is used to measure the antenna, as shown in Figure 10b. Figure 11 shows the measured and simulated return loss of the proposed antenna, where reflection coefficients below −10 dB from 4.83 to 6.1 GHz can be obtained, and the fractional bandwidth is about 23%. Moreover, the simulation and measurement agree with each other.

### 4.2. Broadside Gain

**Figure 12:**
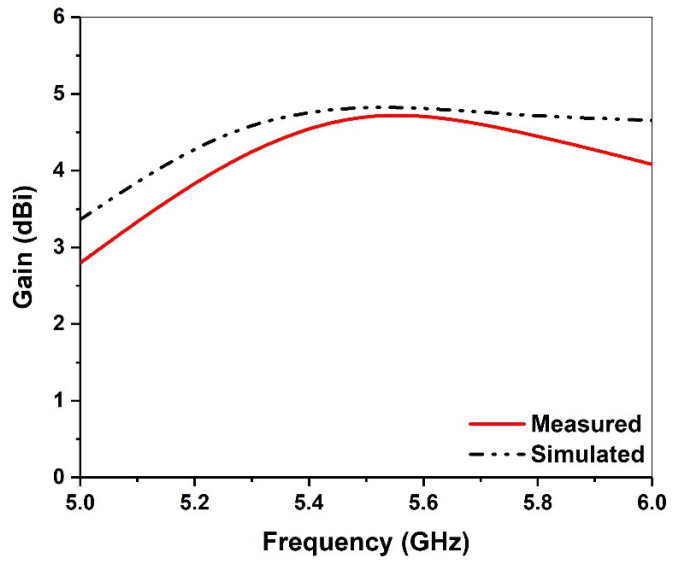
Simulated and measured broadside gain.

Figure 12 shows the measured and simulated gains over the entire frequency range of the proposed antenna. The first resonant frequency is close to 5.25 GHz, and the overall radiation structure is similar to that of the PIFA antenna with small attenuation, long transmission distance, large coverage and omnidirectional characteristics. The second resonant frequency is close to 5.6 GHz, which is excited by the upper patch. The transmission distance is short and the attenuation is large, so high gain is needed to enhance.

### 4.3. Radiation Pattern

**Figure 13:**
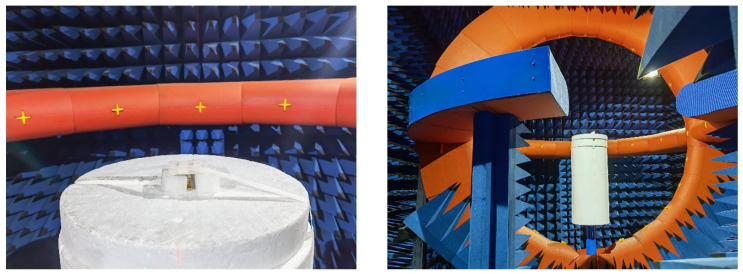
Measurement photograph in an anechoic chamber.

**Figure 14:**
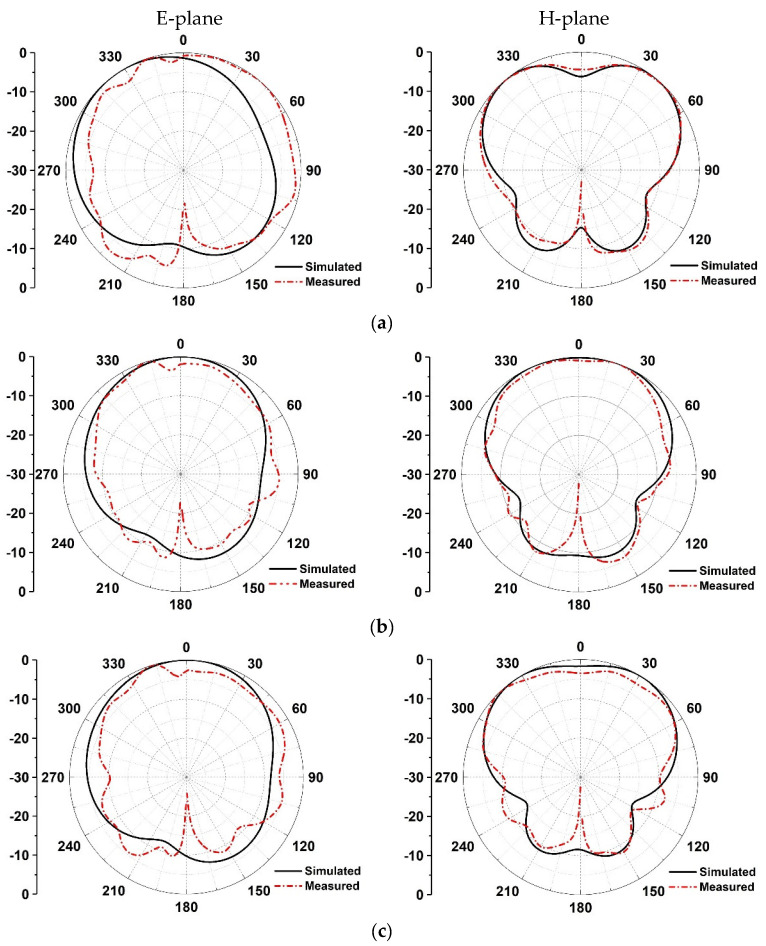
Simulated and measured 2D radiation pattern of proposed antenna with different resonant frequencies: (**a**) 5.25 GHz, (**b**) 5.5 GHz, (**c**) 5.75 GHz.

The radiation patterns of the proposed antenna are measured in an anechoic chamber (see Figure 13), and corresponding results are shown in Figure 14. It can be seen that when the microstrip antenna is folded, the two-dimensional radiation pattern of the E-plane is slightly inclined, but has good omnidirectional characteristics as a whole, and large coverage. From the perspective of the H-plane, the two-dimensional radiation pattern of the folded antenna maintains good symmetry and exhibits horizontal omnidirectional characteristics around 45°. The simulations are in good agreement with the measurement.

### 4.4. Comparison Table and Application

To provide a better comparison, the performances of the proposed antenna and other existing works are summarized in Table 2. The gain of the antenna presented in [1,21,22] is better than that of the proposed antenna, but it also has the disadvantage of large physical size and inferior fractional bandwidth, as shown in Table 2. Although the antenna proposed in [23,24] has smaller dimensions, it can be observed that the fractional bandwidth of the designed antenna is relatively larger than that reported by the authors, and the gain is comparable. Therefore, it is found that the proposed antenna has the advantages of small size and excellent fractional bandwidth within acceptable gain, indicating its promising application in wireless communication.

## 5. Conclusions

A double-layer planar microstrip antenna is proposed and measured in this paper. The impedance bandwidth of the proposed antenna is from 4.83 to 6.1 GHz, and a fractional bandwidth of 23% is obtained. When maintaining a wide bandwidth, we adopt the principle of folding at the zero point of the electric field, then the size is reduced by half to the compact size of only 25 × 40 × 6 mm^3^. Moreover, the overall structure has PIFA radiation characteristics for realizing omnidirectional communication. Through optimizing parameters and varying the location of the feeding point, good impedance matching and wide bandwidth can be obtained. The measured results are in good agreement with the simulated results, which indicates that this antenna can be considered a potential choice for global home portable wireless network coverage.

## Figures and Tables

**Table 1:** Optimized values of the designed antenna.

Parameter	*L_GND_*	*W_GND_*	*Lsub*	*Wsub*	*L*	*W*	*La*	*Wa*	*Lb*	*Wb*	*Lx*	*Wy*	*h*	*Xf*
Unit (mm)	40	25	35	12	17.2	20	9.9	0.9	2.9	3.2	6.6	8	6.1	−5.4

**Table 2:** Comparison of the proposed antenna with the existing works in the literature.

Ref.	Physical Size (mm^2^)	Electrical Size (λ_0_^2^)	Bandwidth (GHz)	Gain (dBi)
[1]	40 × 60	0.73 × 1.1	15.3% (5.05–5.85)	7.5
[21]	54 × 100	1 × 1.8	2.5% (5.725–5.850)	9
[22]	28 × 97	0.51 × 1.7	9.7% (5.49–6.024)	5.23
[23]	19.5 × 10	0.36 × 0.2	13% (5.15–5.85)	4.1
[24]	29.5 × 21	0.55 × 0.39	4.7% (5.27–5.53)	n/a
This work	25 × 40	0.45 × 0.73	23% (4.83–6.1)	4.7
